# Split notochord syndrome associated with dorsal neuroenteric fistula: A rare entity

**DOI:** 10.4103/1817-1745.76112

**Published:** 2010

**Authors:** Punit Srivastava, A. N. Gangopadhyay, D. K. Gupta, S. P. Sharma

**Affiliations:** Department of Pediatric Surgery, IMS, BHU, Varanasi, Uttar Pradesh, India

**Keywords:** Dorsal enteric fistula, split notochord, syndrome

## Abstract

Split notochord syndrome (SNS) is an extremely rare congenital malformation associated with anomalies of the vertebral column, gastrointestinal tract and central nervous system. Twenty cases of SNS associated with dorsal enteric fistula have been reported in literature till date. The present report describes a unique case of SNS associated with lumbosacral meningomyelocele, dorsal neuroenteric fistula and dorsal herniation of right kidney along with vessels. The neonate was well managed by excision of enteric fistula, closure of duramater of meningomyelocele and repair of posterior wall hernia after placement of kidney in renal fossa. This kind of entity is uncommon and not been reported earlier.

## Introduction

Split notochord syndrome (SNS) is frequently associated with vertebral anomalies.[1] Complete cleft of the vertebral column is associated with gastrointestinal tract and central nervous system anomalies. It is an extremely rare form of spinal dysraphism.[2–4] Management of SNS associated with dorsal enteric fistula varies from case to case, depends upon the associated anomalies and system involved; therefore, management must be individualized. It is also considered that staging procedure is needed for proper correction of these anomalies.[5,6]

## Case Report

**Figure 1:**
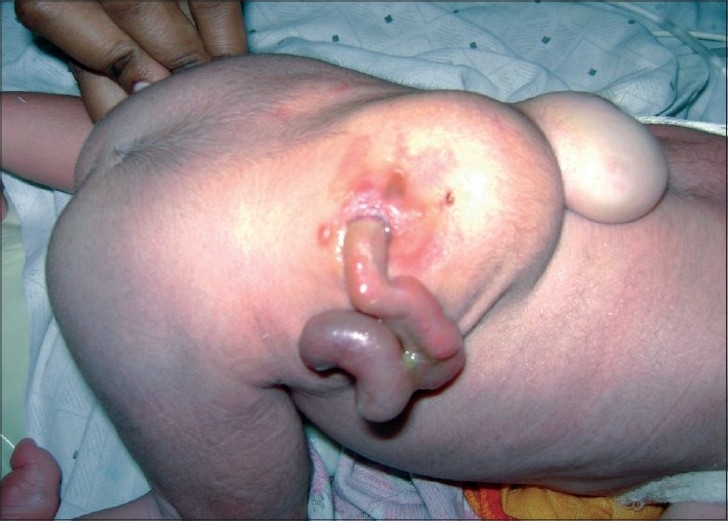
Neonate having meningomyelocele with dorsal neuroenteric fistula

**Figure 2:**
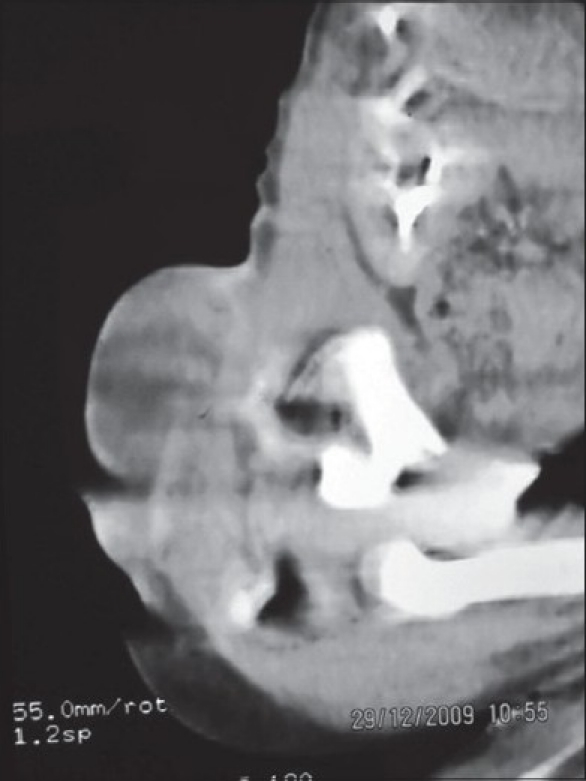
CT scan suggests gap in vertebra with sac containing soft tissue

First born, 1-day-old, 2.5 kg, male baby was admitted in the Department of Pediatric Surgery, with a swelling in lumbosacral region along with an intestine like structure overlying it. No other obvious congenital anomalies were detected. The antenatal history was uneventful. There was no history of drug intake, fever with rash and radiation during antenatal period. Baby was born on full-term, normal vaginal delivery. On clinical examination, there was a cystic swelling, well covered with skin, over lumbosacral area (meningomyelocele) and an intestine like structure was present at the summit of swelling (dorsal enteric fistula)[Figure 1]. There was also a paraspinal muscular defect (hernia) just superior to the swelling, which contained some solid tissue. The baby was passing meconium and urine normally and was moving both his lower limbs normally without any neurological deficit. Plain X-ray of the lumbosacral spine suggested complete splitting of lumbar vertebrae and sacrum. There was no sign of hydrocephalus on cranial ultrasonography. Computed tomography (CT) scan of abdomen and spine[Figure 2] revealed complete splitting of lower lumbosacral vertebrae, dorsal enteric fistula and posterior herniation of kidney as well.

Peroperative finding was meningomyelocele along with dorsal enteric fistula which was non-communicated and attached to the sigmoid mesocolon. There was also paraspinal lumbar hernia which contained kidney and upper ureter.

Neonate was well managed by excision of dorsal enteric fistula, closure of spinal defect and repair of posterior muscle wall after placement of kidney in retroperitoneal renal fossa as a single-stage procedure.

## Discussion

The SNS, as proposed by Bentley and Smith,[7] (also known as posterior spina bifida, combined spina bifida, neurenteric fistula, and dorsal enteric fistula) is an extremely rare form of dysraphism (less than 25 cases have been described in the literature so far). It was first described by Rembe in 1887.[8] Only one case was discovered in an adult and others were in newborn and young children and were reported both in males as well in females.[9] It is a complex malformation, comprising vertebral anomalies (anterior and posterior spina bifida, butterfly vertebrae), central nervous system abnormalities (diastematomyelia, diplomyelia, myelomeningocele) and intestinal anomalies (fistulas, dermal sinus tract, diverticula and enteric cysts). The syndrome manifests as a cleft in the dorsal midline of the body through which intestinal segments are exteriorized (often with an associated fistula), myelomeningocele, and occasionally as a teratoma. The hydrocephalus and diastematomyelia/diplomyelia are a frequent association. However, babies do not necessarily present with functional spinal cord defects; in some reported cases, the motor function of the lower limbs is normal.

Currently, the most widely accepted theory suggests a primary notochord defect (the notochord is split, but not completely separated from the primitive intestine), resulting in secondary changes to the paraxial mesoderm, which is responsible for the formation of the spinal column, giving rise to a medial interosseous space. Through this space, the endoderm and the underlying primitive intestine herniate, subsequently adhere to the dorsal ectoderm, and eventually rupture out.[10,11] This way, the SNS represents an extreme end of the spectrum of neuroenteric cyst.[11]

The dorsal enteric fistula is due to persistent connection between the endoderm and ectoderm, resulting in splitting of the notochord. The fistula traverses the prevertebral soft tissue, the vertebral bodies, and the spinal canal with its contents. Any portion of this tract may involute or become fibrous, leading to a fistula or a cyst. The dorsal enteric sinus, a remnant of the posterior portion of the tract, has an opening on the skin surface. Dorsal enteric cysts are trapped remnants of the middle portion of the tract, found in the intraspinal or paraspinal compartments. The dorsal enteric diverticulum is a tubular diverticulum arising from bowel and represents a remnant of the anterior portion of the tract. Patients with dorsal enteric fistula present as a newborn with a bowel ostium exposed on the back. Intraspinal enteric cysts usually present between 20 and 40 years as episodic local or radicular pain that may progress to myelopathic symptoms. The location of the intestinal fistula may vary from case to case, and may be found either in the distal ileum/cecum or in the large intestine (most of the cases). The disease affects both males and females, and there exists a high incidence of urogenital malformations and anorectal malformations.

Antenatal sonographic findings depend on the lesion and the associated anomalies and may suggest prenatal diagnosis of SNS in some cases.[12] Preoperative investigations are needed to identify and delineate the extent of the disease. It includes plain X-ray of the spine, ultrasonography and CT scan of the abdomen, head and spine. A magnetic resonance imaging study may be needed for further evaluation of the case. Other investigations like fistulography, micturating cystourethrography (MCU), etc., are needed in some cases.[5] Management of SNS associated with dorsal enteric fistula varies from case to case as the associated anomalies and the systems involved vary. It is also considered that staging procedure is needed for proper correction of the anomalies.[6] Our case was unique in it was managed by a single-stage procedure comprising excision and repair of meningomyelocele, disconnection of the dorsal enteric fistula along with repair of posterior abdominal wall hernia. The posterior paraspinal hernia was due to weakness of paravertebral muscles. The baby was well in 1-month follow-up. The urodynamic study, MCU and neurological evaluation are still needed in further follow-ups. Few survivals of cases with SNS have been reported but the overall prognosis of a patient with SNS depends on the associated anomalies and extent of the lesions.[6]
